# Lower IgA Levels in Chronic Spontaneous Urticaria Are Associated With Lower IgE Levels and Autoimmunity

**DOI:** 10.3389/fimmu.2021.657211

**Published:** 2021-05-03

**Authors:** Merle Sauer, Jörg Scheffel, Stefan Frischbutter, Pavel Kolkhir, Yi-Kui Xiang, Frank Siebenhaar, Sabine Altrichter, Marcus Maurer, Martin Metz, Karoline Krause

**Affiliations:** ^1^Dermatological Allergology, Allergie-Centrum-Charité, Department of Dermatology and Allergy, Charité-Universitätsmedizin Berlin, Berlin, Germany; ^2^Berlin Institute of Health (BIH) at Charité – Universitätsmedizin Berlin, Berlin, Germany; ^3^Division of Immune-Mediated Skin Diseases, I.M. Sechenov First Moscow State Medical University (Sechenov University), Moscow, Russia

**Keywords:** immunoglobulin A, immunoglobulin E, chronic spontaneous urticaria, autoreactivity, autoimmune disease, basophils, eosinophils

## Abstract

**Background:**

The pathogenesis of chronic spontaneous urticaria (CSU) is still insufficiently understood. Recent findings suggest that immunoglobulins, in particular IgE but also IgA, play a role in the development of CSU.

**Objective:**

Our aim was to assess differences in clinical and laboratory markers between CSU patients with and without lower levels of serum IgA and IgE.

**Methods:**

We analyzed the data of 606 patients with CSU by dividing them into four groups based on their IgA and IgE levels. The groups were compared for their spectrum of symptoms, disease activity, concomitant autoimmunity and routine laboratory markers. Autoreactivity was assessed by basophil activation test (BAT). Moreover, IgE-anti-thyroid peroxidase (TPO) was measured.

**Results:**

Of the patients with lower IgE levels, 66.5% also had lower IgA levels (r=0.316, p<0.001). Patients with lower IgA and lower IgE levels showed a higher prevalence of recurrent angioedema (p=0.03, p=0.04) and concomitant autoimmunity (p=0.006, p<0.001). Autoreactivity was also found more frequently in patients with lower IgA and lower IgE levels (p=0.003, p<0.001). Reduced basophil counts were linked to both, lower IgA and lower IgE levels (p<0.001), whereas low eosinophil counts were primarily present in patients with lower IgE levels (p=0.04, p<0.001). Patients with elevated IgE-anti-TPO levels had lower IgA (p=0.007) and IgE levels (p=0.001).

**Conclusion:**

Lower IgA levels in CSU are linked to lower IgE levels and features of autoimmune urticaria. Our findings encourage to screen CSU patients for serum IgA and IgE levels and to further assess their role as disease biomarkers.

## Introduction

Chronic spontaneous urticaria (CSU) is a common disease that affects around 1% of the general population (1). It is characterized by the appearance of pruritic wheals, angioedema or both for more than six weeks without a specific and definite triggering factor (2).

The symptoms of CSU are primarily caused by the activation and degranulation of skin mast cells (3). The specific mechanisms that lead to this activation are still not clarified entirely. Based on recent pathomechanistic studies and different response profiles to anti-immunoglobulin E (IgE) treatment, the concept of two different types of autoimmunity in CSU was established (4). In type I autoimmunity, or autoallergy, patients have IgE-antibodies against different autoantigens, such as thyroid peroxidase (TPO) (5), interleukin-24 (6) or double stranded DNA (7) that crosslink the IgE on mast cells and basophils and thus lead to degranulation. Type IIb autoimmunity is characterized by mast cell-activating IgG-antibodies, for example against IgE (8) or its high-affinity receptor FcϵRI (9), that induce histamine release of mast cells and basophils. Patients with type IIb autoimmunity are characterized by a positive autologous serum skin test (ASST), serum autoreactivity in basophil activation test (BAT) or basophil histamine release assay (BHRA) and a positive immunoassay for IgG-antibodies against FcϵRI or IgE (10). However, recent findings show that only in 8% of patients with type IIb autoimmune CSU all three elements are present simultaneously (11).

Low levels of IgE in patients with CSU have been linked to type IIb autoimmunity (11). Also, total levels of IgE are linked to the response to treatment with omalizumab, a therapeutic anti-IgE-antibody, with non-responders to this third-line treatment showing lower levels of IgE (12). Patients with type IIb autoimmune CSU also respond slower to omalizumab treatment (13). In contrast, CSU patients with type I autoimmunity and high IgE levels show better and faster responses to omalizumab, respectively (14, 15).

Currently, little is known about the role of immunoglobulin A (IgA) in CSU. Selective immunoglobulin A-deficiency (SIgAD) is the most common primary immunodeficiency. It is defined as a serum IgA level of less than 0.07 g/l with normal levels of serum immunoglobulin G (IgG) and immunoglobulin M (IgM) in patients older than four years in whom other causes of hypogammaglobulinemia have been excluded (16). Patients with SIgAD have a higher risk of autoimmune diseases (17), as do patients with CSU (18). Furthermore, in patients with SIgAD the prevalence of inflammatory skin diseases including CSU is higher (19). Interestingly, Frossi et al. found SIgAD in patients with CSU to be linked to the presence of other autoimmune diseases and signs of type IIb autoimmune CSU, such as positive ASST and BAT results (20). However, only four patients (all of them with exceptionally low IgA levels) were included in this study. Therefore, further work on the importance of IgA in CSU in a bigger cohort is required.

We hypothesized that lower levels of IgA are linked to features of type IIb autoimmune CSU including lower IgE. To test our hypothesis, we measured total serum IgA and IgE levels in more than 600 CSU patients and assessed those with lower levels of one or both for their clinical and laboratory features.

## Methods

### Patients

We analyzed the data of 606 patients with CSU treated at the Urticaria Reference and Excellence Center (UCARE) (21) at Charité-Universitätsmedizin Berlin. All patients provided oral and written informed consent that included the publication of their pseudonymized data. For affected children (n=10), parents provided written informed consent. The study was approved by the Ethics Committee of Charité-Universitätsmedizin Berlin, Germany.

The patients were divided into four groups based on their IgA and IgE levels: 1) lower IgA and lower IgE levels (IgA^lower^IgE^lower^), 2) lower IgA and normal or elevated IgE levels (IgA^lower^IgE^n/hi^), 3) lower IgE and normal or elevated IgA levels (IgA^n/hi^IgE^lower^) and 4) normal or elevated IgA and IgE levels (IgA^n/hi^IgE^n/hi^). Based on the median IgA value of 1.84 g/l in this cohort of 606 CSU patients, we classified patients who had a serum IgA level of <1.84 g/l as having lower IgA levels. For lower IgE levels, the cut-off of <40 kU/l was used as this had previously been reported to be clinically relevant in CSU patients (11, 22).

All patients with complete routine diagnostic data on immunoglobulin levels A, E, G and M, were included in the study (n=523). Additionally, to increase group sizes, partially missing immunoglobulin values were determined in retrospect from frozen sera of patients who were known to have either lower IgA or lower IgE levels (n=83).

Patients treated with anti-IgE-antibodies (omalizumab) or immunosuppressives were not included in the study. Moreover, patients were asked, if possible, to stop taking oral corticosteroids and antihistamines, starting four weeks before the analysis, to ensure reliable results.

### Clinical Markers

Disease activity was measured by the 7-day once-daily urticaria activity score (UAS7) that is based on wheal number and itch severity documented by the patient (minimum: 0, maximum: 42) (2). To assess health-related quality of life impairment, the dermatology life quality index (DLQI) was applied (minimum: 0, maximum: 30) (23). Furthermore, the spectrum of symptoms (wheals with or without angioedema) and the prevalence of concomitant diseases, especially autoimmunity, were assessed.

### Routine Laboratory Markers

Aside from serum levels of IgA and IgE, different serologic markers were assessed in all patients including C-reactive protein, leukocyte, basophil and eosinophil count, IgG and IgM levels.

### Assessment of Autoimmunity

Patients were screened for the presence of comorbid autoimmune disease and elevated levels of anti-neutrophil cytoplasmic antibodies (ANCA), (cANCA ≥10 U/ml, pANCA ≥5 U/ml), anti-nuclear antibodies (ANA) (≥1:160), rheumatoid factor IgM (≥20U/ml), circulating immune complexes (≥55 µg/ml), IgG-anti-TPO (≥35 kU/l) or thyroid stimulating hormone-receptor-antibodies (≥2 U/l).

### Assessment of Autoreactivity

Autoreactivity was assessed by an indirect BAT to screen for autoantibodies in the patients’ serum. Moreover, IgE-anti-TPO as a potential relevant autoallergen in CSU was measured (5).

The BAT was performed with fresh healthy donor basophils and frozen patient serum that was thawed and diluted in phosphate-buffered saline (PBS) (final concentration of 20%). 50 µl of heparinized whole blood, taken from the same healthy donor for all experiments, was incubated with 50 µl of 20% serum for 15 minutes at 37°C and with 5% CO_2_ in a 96 well plate. As a positive control, 50 µl PBS containing 2.5 µg/ml mouse anti-human IgE HP6029 and 2.5 µg/ml mouse anti-human IgE HP6061 (both SouthernBiotech, Birmingham, AL) was used. PBS functioned as negative control. After washing the samples with 150 µl PBS + 2mM ethylenediaminetetraacetic acid (EDTA; Invitrogen, Carlsbad, CA) the plate was centrifuged at 340 x g for 10 minutes at 4°C. The supernatant was aspirated with a vacuum pump and the cells were stained with antibodies against CD3 (BD Biosciences, Franklin Lakes, NJ, REF#560365), CD193 (BD Biosciences, Franklin Lakes, NJ, REF#558208), CD63 (BD Biosciences, Franklin Lakes, NJ, REF#561982) and CD203c (Beckman Coulter, Brea, CA, REF#IM3575) at 1:42 each in FACS buffer (1% bovine serum albumin [BSA; Serva, Heidelberg, Germany] in PBS) supplemented with 2.4% Kiovig (Baxter AG, Vienna, Austria). The cells were lysed with 200 µl of red blood cell lysis buffer (BioLegend, San Diego, CA) for five minutes. After centrifugation, the supernatant was discarded and the pellet was resuspended in 250 µl MACS buffer (1% BSA + 2 mM EDTA in PBS). Samples were then measured by flow cytometry (MACS Quant, Miltenyi Biotec, Bergisch Gladbach, Germany) and analyzed using FlowJo (v.10.6.1, BD Biosciences, Franklin Lakes, NJ). The gating strategy used here is shown in the **Supplemental Figure 1**. The BAT was considered positive, if more than 7.77% of the total basophils were both CD63 and CD203c positive. As cut-off, the 95th percentile of double positive cells induced by control sera (n=51) was used.

CSU serum levels of IgE-anti-TPO were measured in an enzyme-linked immunosorbent assay. The plate was coated overnight with 2 µg/ml of MHE-18 (BioLegend, San Diego, CA). After blocking with 1% fetal bovine serum (FBS, Biochrom, Berlin, Germany) in tris-buffered saline (TBS), plates were incubated with serum (diluted 1:5 in TBS, overnight, at 4°C), followed by biotinylated human-TPO (in.vent Diagnostica GmbH, Hennigsdorf, Germany). For detection, streptavidin-HRP (ThermoFisher Scientific, Waltham, MA) and ECL Prime (GE Healthcare, Chicago, IL) were used. Between each step, intensive washing with TBS containing 0.05% Tween 20 was performed. Chimeric human IgE-anti-TPO was used as positive control and standard. It was obtained from the supernatant of SP-2/Sp1.4 transfected mouse myeloma cells (kindly provided by Sandra McLachlan, Thyroid Autoimmune Disease Unit, Cedars-Sinai Medical Center and University of California Los Angeles) grown in 2 mM L-Glutamine, 10% FBS gold IMEM-medium, 10 µg/ml Streptomycin and 100 U/ml Penicillin (all Sigma-Aldrich, Deisenhofen, Germany), as described before (5). IgE-anti-TPO levels >1.09 ng/ml were considered as elevated. As cut-off, the 95th percentile of IgE-anti-TPO levels measured in healthy control sera (n=14) was used.

### Statistical Analysis

Statistical analysis was performed with the Statistical Package for the Social Science (IBM SPSS version 27; IBM Corp, New York, NY) and figures were created using GraphPad Prism (version 9.0.0; GraphPad Software, San Diego, CA). Normal distribution was tested with Kolmogorov Smirnov test. As the data was not normally distributed, the median (Md) and interquartile range (IQR) were used. For comparison of continuous variables, the Mann-Whitney-U test was performed when comparing two groups and the Kruskal-Wallis test (one-way ANOVA) was applied when comparing more than two groups. Correlation was assessed using Spearman-Rho test for variables that were not normally distributed. Binary variables were analyzed using Pearson Chi-square test. Post hoc analysis involved pairwise comparisons using the z-test of two proportions with a Bonferroni correction. Statistical significance was indicated by p ≤ 0.05.

## Results

### Lower IgA Levels Are Linked to Lower IgE and IgG Levels and Higher IgM Levels

In our study cohort the median age of patients was 43 years, ranging from 13 to 82 years. More female (n=461) than male patients (n=145) with CSU were included in the cohort. The median disease duration was two years and the median age at diagnosis 36 years. During the examination period, 4.7% of patients (n=17) received low to medium doses of oral corticosteroids and 23.4% (n=88) received antihistamines (**Table 1**).

**Table 1:** Main clinical and laboratory features of the patients.

	Lower IgA and lower IgE levels(IgA^lower^ IgE^lower^)	Lower IgA and normal or elevated IgE levels (IgA^lower^ IgE^n/hi^)	Lower IgE and normal or elevated IgA levels (IgA^n/hi^ IgE^lower^)	Normal or elevated IgA and IgE levels (IgA^n/hi^ IgE^n/hi^)	Signifi-cance level
	n=149 (24.6%)	n=154 (25.4%)	n=75 (12.4%)	n=228 (37.6%)	
Female patients, % (n)	86.6 (129)	78.6% (121)	73.3 (55)	68.4 (156)	**p=0.001**
Age at diagnosis in years, md (IQR)	40.00 (26.00-49.60)	31.92 (23.00-42.00)	41.00 (27.25-50.00)	37.00 (26.42-50.00)	**p=0.001**
Disease duration in years, md (IQR)	1.58 (1.00-5.00)	2.08 (1.00-7.00)	2.00 (1.00-6.00)	2.14 (1.00-8.00)	p=0.16
Antihistamine treatment*, % (n)	23.7 (23)	23.7 (22)	25.5 (14)	22.1 (29)	p=0.97
Systemic corticosteroid treatment*, % (n)	3.2 (3)	7.9 (7)	5.7 (3)	3.1 (4)	p=0.35
IgA in g/l (0.7-4.0), md (IQR)	1.27 (0.87-1.53)	1.42 (1.13-1.61)	2.37 (1.98-3.16)	2.47 (2.14-3.10)	**p<0.001**
IgE in kU/l (0.0-100.0), md (IQR)	14.60 (6.37-23.60)	122.50 (64.00-241.00)	17.20 (8.54-27.70)	160.00 (89.95-287.00)	**p<0.001**
IgG in g/l (7.0-16.0), md (IQR)	9.05 (7.81-10.80)	9.57 (8.51-10.52)	10.05 (8.83-11.74)	10.24 (8.77-12.07)	**p<0.001**
IgM in g/l (0.4-2.3), md (IQR)	1.05 (0.69-1.48)	0.99 (0.67-1.40)	0.98 (0.71-1.30)	0.97 (0.67-1.37)	p=0.33
UAS7 (0-42), md (IQR)	19.00 (10.00-27.00)	18.00 (12.00-25.00)	17.00 (9.00-29.00)	16.00 (8.00-23.00)	p=0.14
DLQI (0-30), md (IQR)	9.00 (4.00-13.00)	9.50 (6.00-15.00)	6.00 (3.00-12.50)	6.00 (3.00-11.00)	**p=0.01**
Presence of angioedema, % (n)	49.7 (73)	33.8 (50)	34.3 (24)	35.1 (74)	**p=0.01**
C-reactive protein in mg/l, md (IQR)	2.00 (1.00-5.40)	1.90 (0.70-6.45)	2.25 (0.90-5.25)	2.50 (1.00-5.60)	p=0.65
Leukocyte count/nl, md (IQR)	6.16 (5.15-7.47)	6.61 (5.17-7.85)	5.59 (4.78-7.01)	6.31 (5.43-7.58)	p=0.12
Eosinophil count/nl, md (IQR)	0.09 (0.06-0.17)	0.17 (0.09-0.29)	0.13 (0.08-0.19)	0.16 (0.10-0.24)	**p<0.001**
Basophil count/nl, md (IQR)	0.01 (0.00-0.02)	0.02 (0.01-0.03)	0.02 (0.01-0.03)	0.02 (0.01-0.04)	**p<0.001**
IgE-anti-TPO in ng/ml, md (IQR)	1.01 (0.80-1.17)	0.91 (0.73-1.16)	1.00 (0.83-1.13)	0.92 (0.76-1.08)	p=0.26

DLQI, Dermatology life quality index; IgA, Immunoglobulin A; IgE, Immunoglobulin E; IgG, Immunoglobulin G; IgM, Immunoglobulin M; IQR, Interquartile range; Md, Median; TPO, Thyroid peroxidase; UAS, Urticaria activity score.

*Treatment with antihistamines or systemic corticosteroids (prednisone, median 7 mg/d, range 1-40 mg/d) at the time of immunoglobulin assessment or in the 7 days before.

Reference values for serum immunoglobulin classes are indicated in brackets. For clinical scores minimum and maximum values are presented. Significances for differences between the groups were measured by Kruskal-Wallis test for continuous variables and Pearson Chi-square test for binary variables. Statistically significant correlations are written in boldface.

**Figure 1:**
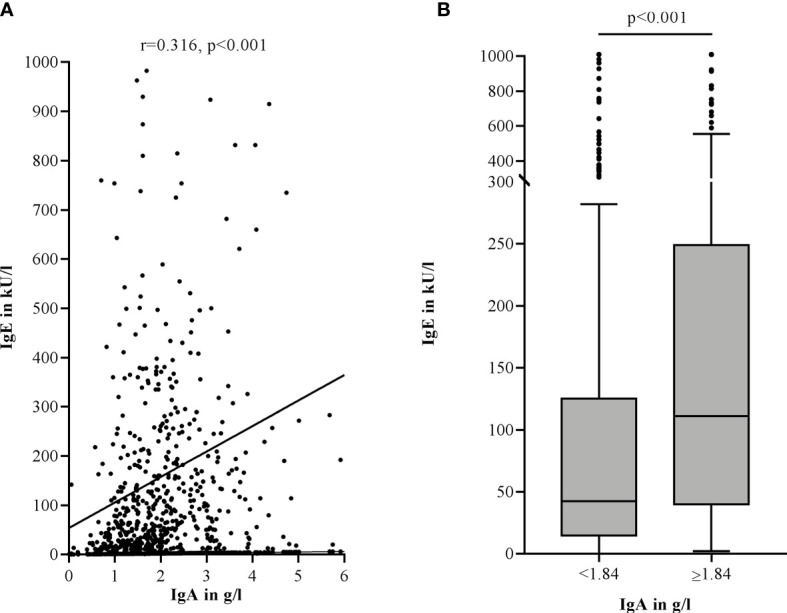
Lower IgA levels are correlated with lower total IgE levels in the serum of CSU patients (Spearman-Rho) **(A)**. Lower IgA levels are associated with lower total IgE levels. Boxes are displayed as median and interquartile range. The whiskers indicate the range. For statistical significance, a Mann-Whitney-U test was performed **(B)**. IgA levels >6 g/l **(A)** and IgE levels >1000 kU/l **(A, B)** are not shown in this graphic.

More than a third of CSU patients showed lower IgE levels (n=224, 37.0%). Of these patients, 66.5% (n=149) were classified as having lower IgA levels (**Table 1**). IgA and IgE levels were significantly correlated (r=0.316, p<0.001) (**Figure 1A**) and patients with lower IgA (<1.84 g/l) also had significantly lower IgE levels (md=42.50 kU/l *vs.* md=111.00 kU/l, p<0.001) (**Figure 1B**).

**Figure 2:**
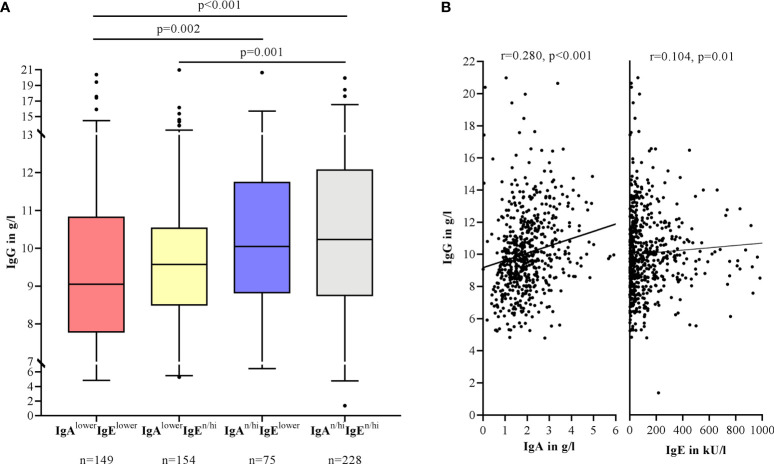
Lower IgG levels are associated with lower IgA levels in the serum of CSU patients. Boxes are displayed as median and interquartile range. The whiskers indicate the range. For statistical significance, a Kruskal-Wallis test was performed **(A)**. Correlation of IgG with IgA and IgE levels by Spearman-Rho. IgA levels >6 g/l and IgE levels >1000 kU/l are not shown in this graphic **(B)**.

Moreover, IgG levels were particularly lower in patients with lower IgA levels. The difference was significant between IgA^lower^IgE^lower^ patients compared to IgA^n/hi^IgE^lower^ patients (p=0.002) and IgA^n/hi^IgE^n/hi^ patients (p<0.001) (**Figure 2A**). IgG levels also correlated significantly with IgA (r=0.280, p<0.001) and less strongly with IgE levels (r=0.104, p=0.01) (**Figure 2B**).

IgM levels revealed no significant differences between the groups but were especially high in IgA^lower^IgE^lower^ patients (**Table 1**) and showed a weak negative correlation with IgA levels (r=-0.090, p=0.03).

### Lower IgA and IgE Levels Are Associated With Female Gender, High Rates of Angioedema and Lower Basophil and Eosinophil Counts

Levels of IgA were lower in women (md=1.76 g/l) compared to men (md=2.12 g/l) (p<0.001), as were levels of IgE (md=60.90 kU/l *vs.* 118.00 kU/l, p<0.001). The proportion of females was significantly higher in IgA^lower^IgE^lower^ patients compared to IgA^n/hi^IgE^n/hi^ patients (86.6% *vs.* 68.4%, p<0.001). With a median age of 32 years at diagnosis, IgA^lower^IgE^n/hi^ patients were significantly younger compared to all other groups (IgA^lower^IgE^lower^: p=0.006, IgA^n/hi^IgE^lower^: p=0.009, IgA^n/hi^IgE^n/hi^: p=0.002) (**Table 1**).

The prevalence of recurrent angioedema was highest in IgA^lower^IgE^lower^ patients. Here, nearly half of the patients, namely 49.7% showed angioedema. The difference was significant compared to IgA^lower^IgE^n/hi^ patients (33.8%, p=0.03) and IgA^n/hi^IgE^n/hi^ patients (35.1%, p=0.04) (**Table 1**).

Quality of life impairment, as assessed by DLQI was highest in patients with lower levels of IgA. The difference was significant for IgA^lower^IgE^n/hi^ patients (md=9.5) compared to IgA^n/hi^IgE^n/hi^ patients (md=6.0, p=0.01). The disease activity did not show significant differences between the groups as assessed by UAS7 (**Table 1**). However, for both UAS7 and DLQI, a negative correlation with IgA levels was found (UAS7: r=-0.095, p=0.04; DLQI: r=-0.173, p=0.005). Of note, the correlations increased when only including patients with concomitant recurrent angioedema (UAS7: r=-0.242, p=0.001; DLQI: r=-0.291, p=0.003) and the correlations completely disappeared when excluding patients with concomitant autoimmune phenomena (n=248).

**Figure 3:**
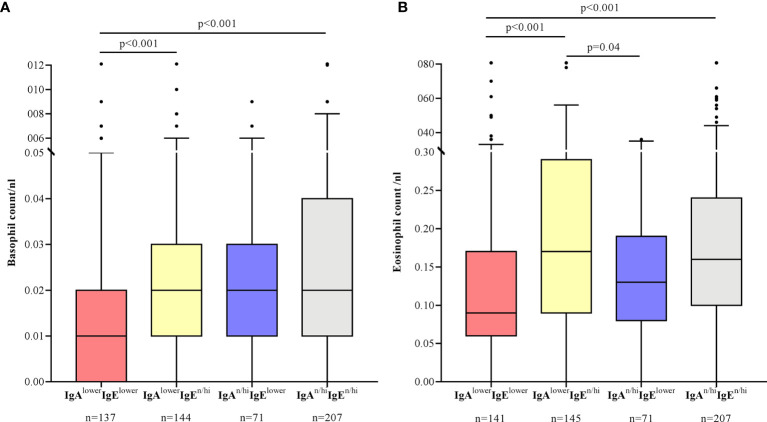
Basophil counts are lowest in CSU patients with lower IgA and IgE levels **(A)**, whereas eosinophil counts are low in CSU patients with lower IgE levels with or without lower IgA levels **(B)**. Boxes are displayed as median and interquartile range. The whiskers indicate the range. For statistical significance, a Kruskal-Wallis test was performed. Basophil counts >0.12/nl and eosinophil counts >0.8/nl are not shown in this graphic.

Basophil counts were lowest in IgA^lower^IgE^lower^ patients. The differences were significant compared to IgA^lower^IgE^n/hi^ patients (p<0.001) and IgA^n/hi^IgE^n/hi^ patients (p<0.001) (**Figure 3A**). Eosinophil counts were mainly linked to lower IgE levels as shown by significantly lower eosinophil numbers in IgA^lower^IgE^lower^ and IgA^n/hi^IgE^lower^ patients compared to IgA^lower^IgE^n/hi^ patients (p<0.001, p=0.04) (**Figure 3B**).

### Lower IgA and IgE Levels Are Linked to High Rates of Concomitant Autoimmune Diseases, Autoreactivity and High Levels of IgE-anti-TPO

**Figure 4:**
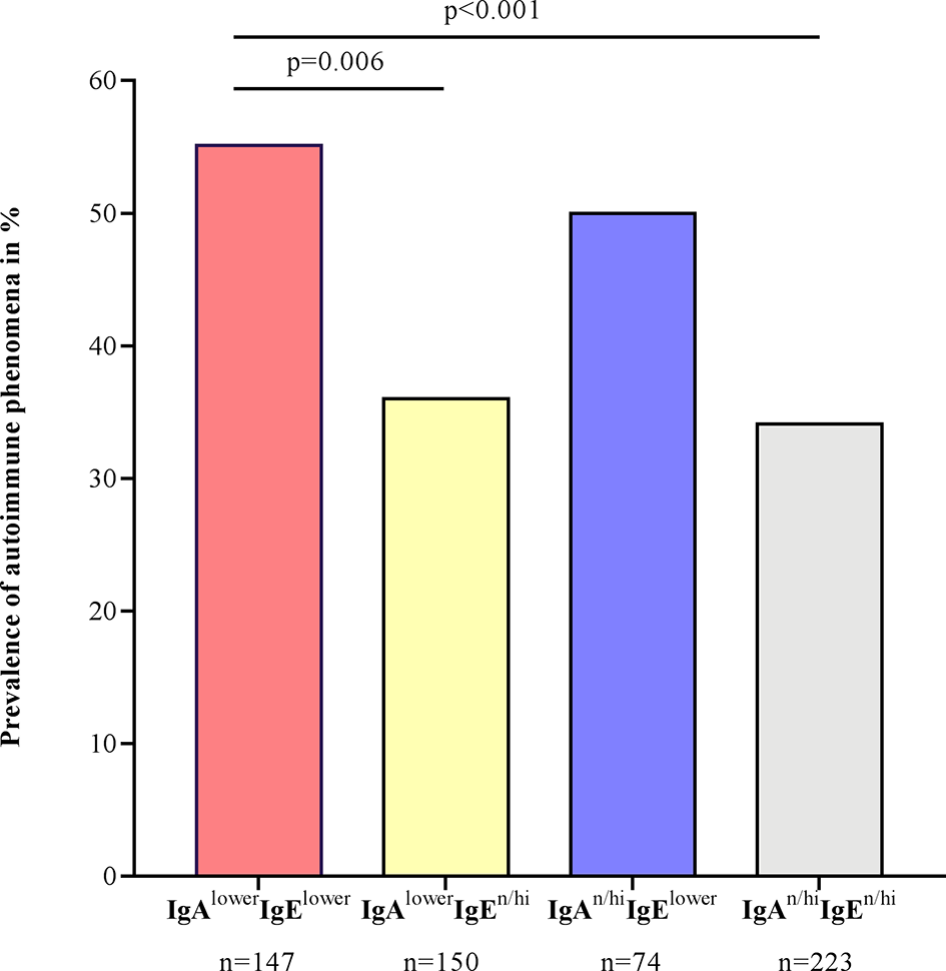
Autoimmune phenomena are more prevalent in CSU patients with lower serum IgE levels. Autoimmune phenomena were defined as the presence of clinically diagnosed autoimmune diseases and/or elevated levels of ANCA, ANA, rheumatoid factor IgM or circulating immune complexes and/of elevated levels of IgG-anti-TPO or thyroid stimulating hormone-receptor-antibodies, if they were in accordance with the clinical symptoms. For statistical significance, the z-test of two proportions with a Bonferroni correction was performed.

Four of ten CSU patients (248 of 594, 41.8%) had one or more comorbid autoimmune disease and/or elevated levels of specific relevant autoantibodies. The most common comorbid autoimmune diseases were Hashimoto’s thyroiditis and Graves’ disease. Positivity to ANA, ANCA, rheumatoid factor IgM or circulating immune complexes was associated mostly to lower IgE levels (IgA^lower^IgE^lower^ and IgA^n/hi^IgE^lower^), whereas the prevalence of thyroid autoimmune diseases was especially high in patients with both lower IgA and lower IgE levels (**Table 2**). In total, the proportion of patients with autoimmune diseases and/or elevated autoantibodies was therefore highest in patients with lower IgE levels (**Figure 4**).

**Table 2:** Prevalence of autoimmune phenomena in patients with CSU sorted by type.

	Type	Criteria	IgA^lower^ IgE^lower^	IgA^lower^ IgE^n/hi^	IgA^n/hi^ IgE^lower^	IgA^n/hi^ IgE^n/hi^	Significance level
**A**	Clinically diagnosed autoimmune disease	Patient history confirmed by treating physician	23.5% (n=35)	14.3% (n=22)	14.7% (n=11)	11.0% (n=25)	**p=0.01**
**B**	ANA, ANCA, rheumatoid factor IgM and/or circulating immune complexes	Positivity of ≥1 of the following	37.0% (n=54)	28.4% (n=42)	34.7% (n=25)	24.7% (n=55)	p=0.06
	ANCA	cANCA ≥10 U/ml, pANCA ≥5 U/ml	6.0% (n=7)	5.6% (n=7)	5.7% (n=3)	4.0% (n=7)	p=0.85
	ANA	≥1:160	38.7% (n=36)	29.1% (n=25)	39.5% (n=17)	24.6% (n=29)	p=0.09
	Rheumatoid factor IgM	≥20U/ml	4.2% (n=6)	6.3% (n=9)	0.0% (n=0)	3.6% (n=8)	p=0.17
	Circulating immune complexes	≥55 µg/ml	11.8% (n=15)	5.6% (n=7)	16.4% (n=10)	8.2% (n=16)	p=0.08
**C**	Thyroid antibodies + clinical phenotype	Positivity of ≥1 of the following + clinic of hypo- or hyperthyroidism	16.4% (n=24)	7.4% (n=11)	8.1% (n=6)	7.0% (n=16)	**p=0.01**
	IgG-anti-TPO	≥35 kU/l	35.0% (n=49)	12.8% (n=18)	16.7% (n=12)	21.4% (n=48)	**p<0.001**
	Thyroid stimulating hormone-receptor-antibodies	≥2 U/l	7.3% (n=10)	12.9% (n=18)	4.2% (n=3)	5.5% (n=12)	p=0.15
	Autoimmune phenomena	**A** and/or **B** and/or **C**	55.1% (n=81)	36.0% (n=54)	50.0% (n=37)	34.1% (n=76)	**p<0.001**

ANA, Anti-nuclear antibodies; ANCA, Anti-neutrophil cytoplasmic antibodies, IgA, Immunoglobulin A; IgE, Immunoglobulin E; IgG, Immunoglobulin G; IgM, Immunoglobulin M; TPO, Thyroid peroxidase. Significances for differences between the groups were measured by Pearson Chi-square test. Statistically significant correlations are written in boldface.

**Figure 5:**
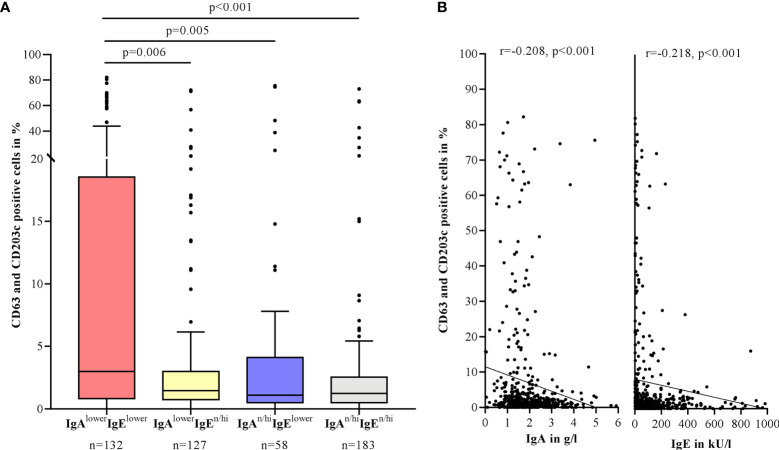
BAT positivity is associated with lower serum IgA and IgE levels in CSU patients. Percentage of CD63 and CD203c positive cells in the BAT after stimulation of healthy basophils with patient serum. Boxes are displayed as median and interquartile range. The whiskers indicate the range. For statistical significance, a Kruskal-Wallis test was performed **(A)**. IgA and IgE levels correlate negatively with the percentage of activated basophils by Spearman-Rho. IgA levels >6 g/l and IgE levels >1000 kU/l are not shown in this graphic **(B)**.

The BAT as a marker of type IIb autoimmunity was positive in 16.6% of CSU patients (n=500), with highest percentages of activated basophils (CD63 and CD203c positivity) in patients with lower IgA and IgE levels (IgA^lower^IgE^lower^). The difference in activation rates was significant compared to all other groups (p=0.006, p=0.005, p<0.001) (**Figure 5A**). This effect was independent of the prevalence of autoimmune diseases as the exclusion of all patients with autoimmune phenomena (n=275 remaining) still revealed highest rates in the IgA^lower^IgE^lower^ group compared to all other groups (p=0.04, p<0.001, p=0.009).

Both IgA and IgE levels showed a significant negative correlation with the percentage of activated cells (IgA: r=-0.208, p<0.001; IgE: r=-0.218, p<0.001) (**Figure 5B**). Positive BAT rates were also found more often in IgA^lower^IgE^lower^ patients (32.6%) compared to IgA^lower^IgE^n/hi^ patients (14.2%, p=0.003) and IgA^n/hi^IgE^n/hi^ patients (7.1%, p<0.001). There was a weak correlation between UAS7 and the percentage of activated cells (r=0.106, p=0.04).

**Figure 6:**
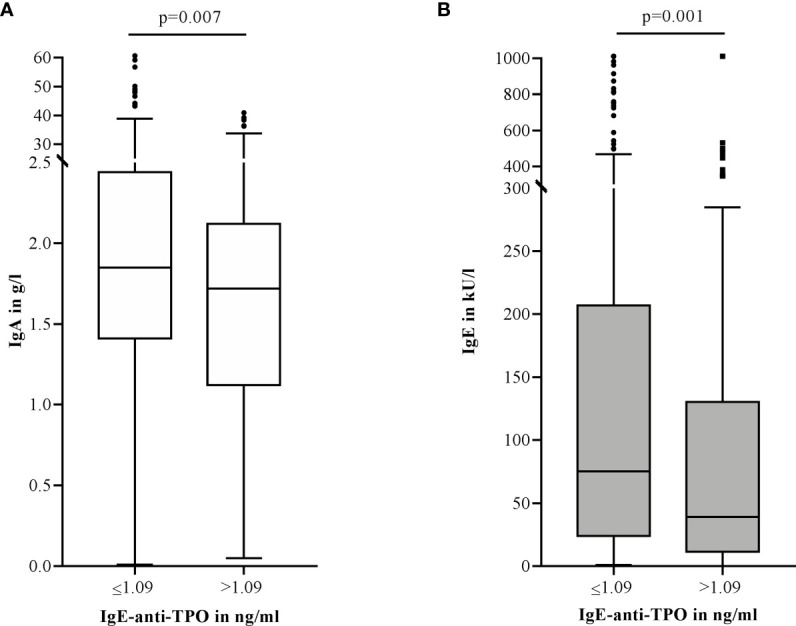
Elevated IgE-anti-TPO levels (>1.09 ng/ml, n=120) in CSU patients are associated with lower serum IgA **(A)** and IgE **(B)** levels compared to patients with normal IgE-anti-TPO levels (≤1.09 ng/ml, n=279). Boxes are displayed as median and interquartile range. The whiskers indicate the range. For statistical significance, a Mann-Whitney-U test was performed. IgA levels >6 g/l and IgE levels >1000 kU/l are not shown in this graphic.

IgE-anti-TPO levels were measured in 399 patients. Although quantitative IgE-anti-TPO levels did not markedly differ between the four groups (**Table 1**), patients with elevated IgE-anti-TPO levels had significantly lower IgA (md=1.72 g/l *vs.* 1.85 g/l, p=0.007) (**Figure 6A**) and IgE levels (md=39.25 kU/l *vs.* 75.40 kU/l, p=0.001) (**Figure 6B**) and IgE-anti-TPO showed a weak negative correlation with IgE levels (r=-0.120, p=0.02). Nearly half of the patients with elevated levels of IgG-anti-TPO or thyroid stimulating hormone-receptor antibodies (n=107), also had elevated IgE-anti-TPO levels (n=52, 48.6%).

## Discussion

This is the first study on IgA levels in a sizeable cohort of CSU patients. Lower IgA and lower IgE levels often appear together and the combination of both is linked to features of autoreactivity (e.g. positive BAT and elevated IgE-anti-TPO levels) and the appearance of autoimmune phenomena.

More than half of the patients with lower IgE levels also showed reduced IgA levels, indicating that these two types of immunoglobulins are linked to each other. This is supported by observations in common variable immunodeficiency, which frequently presents with very low levels of IgA in combination with reduced levels of IgE (24). Interestingly, patients with both lower IgA and lower IgE levels also had reduced IgG levels but increased IgM levels as compared to other groups. An explanation for this finding could be a B-cell defect resulting in an impaired antibody class switch with imbalanced immunoglobulin production in CSU. This has been reported for patients with SIgAD, who showed increased levels of serum IgM as a compensatory mechanism. The increased IgM levels might protect patients against severe infections, as SIgAD patients with normal IgM levels developed severe infections more often (25).

However, it remains to be clarified whether lower IgA and IgE levels were present in patients before the development of their urticaria and were thus involved in the pathogenesis of the disease, or whether the imbalanced immunoglobulins were caused by the disease itself.

A functional role of lower IgA in the pathogenesis of CSU could be attributed to the signaling through FcαRI. In a murine IgE-mediated model of asthma it was shown that stimulation of FcαRI with serum IgA inhibits FcϵRI-induced degranulation of mast cells by IgE based on an antagonism between IgA and IgE *via* their respective receptors (26). In humans, FcαRI is expressed on immune cells such as eosinophils, neutrophils and monocytes but not on mast cells. Serum IgA in humans is supposed to negatively regulate immune cell activation and degranulation *via* FcαRI (27, 28). One could hypothesize that lower IgA in CSU would lead to increased activation of eosinophils, neutrophils and monocytes resulting in inflammation and autoimmunity.

The quality of life was impaired the greatest in patients with lower IgA levels (IgA^lower^IgE^lower^ and IgA^lower^IgE^n/hi^), whereas the CSU disease activity did not differ significantly. We attribute the effect on quality of life to higher rates of concomitant conditions, such as autoimmune phenomena and recurrent angioedema in patients with lower IgA and lower IgE levels. Rates of recurrent angioedema, for instance, were highest in patients with lower IgA and lower IgE levels. Patients with positive ASST results have been found to be more likely to develop angioedema (29). This fits our observation of frequent autoreactivity in patients with lower IgA and IgE levels. Of further interest, Bond et al. reported a link between SIgAD and hereditary angioedema (30).

In our study we found that basophil counts seem to be influenced especially by the combination of lower IgA and lower IgE levels, whereas eosinophil counts are related predominantly to the levels of IgE. Earlier reports found that basopenia is linked to severe, antihistamine-resistant CSU and type IIb autoimmunity (31), which fits our results that patients with lower IgA and IgE levels show more type IIb autoimmunity. It is hypothesized that basopenia is caused by the recruitment of circulating basophils into the wheals (8, 32). Recent findings also indicate a connection between eosinopenia and type IIb autoimmunity, high disease activity, poor response to treatment and lower IgE levels (33). This is in line with the results of our study. Also, it was shown that some CSU patients can benefit from anti-interleukin-5-targeted treatment with mepolizumab (34) or reslizumab (35) which affect recruitment, activation and survival of eosinophils. Beyond this, concomitant lower IgA levels were found in some patients with eosinophil deficiency (36). The exact role of eosinophils in the pathogenesis of CSU, however, remains unclear (37).

Our data imply that lower IgA and lower IgE levels taken together are more sensitive in diagnosing type IIb autoimmunity in CSU patients than lower IgE levels alone. The connection between lower levels of serum IgE and type IIb autoimmunity that we found has been described previously (38, 39). However, the reason for this remains unknown. One hypothesis is that IgE complexes with anti-IgE-antibodies and is therefore not measurable in these patients (40).

Recently, IgM- and IgA-antibodies against the IgE receptor FcϵRI were found in addition to IgG-antibodies, which are characteristic for type IIb autoimmunity in CSU (41). Although only IgM-antibodies could be linked to type IIb autoimmunity so far, based on our results it would be of interest to measure IgA-antibodies in a CSU cohort.

The prevalence of SIgAD was shown to be increased in patients with autoimmune diseases and the other way around (42). Abolhassani et al. proposed that this effect is mediated by a reduced mucosal defence in patients with SIgAD. Thus, antigens from the environment could enter the ciruclation more easily and result in the development of autoreactive antibodies *via* induction of molecular mimicry and cross-reaction with self-antigens (43). In our study only three patients showed signs of SIgAD (meaning an IgA level <0.07 g/l), but it is reasonable to think that lower normal levels of IgA, although not as low as in SIgAD might have a similar, less strong effect. Another explanation for the association of lower IgA levels with autoreactivity may be the presence of IgG- or IgE-anti-IgA-antibodies (44). Since patients with reduced IgA levels are more likely to develop autoreactive antibodies, this could result in the maturation of autoantibodies that are directed against IgE leading to decreased serum IgE levels.

We also found that high IgE-anti-TPO levels, a common autoantibody in type I autoimmunity in CSU, are connected principally to lower total IgE levels. This stands in contrast to earlier findings suggesting that type I autoimmunity in CSU is connected to high levels of IgE (45, 46). Our results, instead, indicate that lower IgE levels may be linked to features of both, type I autoimmunity, as well as type IIb autoimmunity. Of note, we did not assess other specific IgE autoantibodies besides IgE-anti-TPO. As the levels of specific IgE autoantibodies may be more relevant for the diagnosis of type I autoimmunity as compared to the levels of total IgE, future studies should screen for different IgE autoantibodies in order to distinguish between types of autoimmunity in CSU.

A limitation of our study is the classification into lower *vs.* normal and elevated IgA levels based on a median IgA level of 1.84 g/l in our cohort. Whether the distribution of IgA values differs in other populations, needs to be studied. Nevertheless, our results emphasize that not only very low IgA levels are of importance in CSU patients, but also that levels on the lower range of the normal value should be evaluated.

Further limitations in the design of our study are the gender and age differences between the four groups (**Table 1**). The fact that women’s IgA and IgE levels are lower has been found previously (47–49). Therefore, it has to be considered whether sex is a confounding factor in our study. Especially when investigating the prevalence of autoimmune phenomena this could be of relevance, since these are known to be increased in women. But when only looking at the male patients (n=145) in our study population, autoimmune phenomena, although not statistically significant, were also associated with lower levels of IgE (47.4% in IgA^lower^IgE^lower^ and 45.0% in IgA^n/hi^IgE^lower^ patients compared to 31.3% in IgA^lower^IgE^n/hi^ and 27.1% in IgA^n/hi^IgE^n/hi^ patients).

In addition, IgA^lower^IgE^n/hi^ were younger at the time of diagnosis compared to all other groups. IgA levels usually rise with the age (47, 50), which could explain why younger patients had lower IgA levels. However, this effect is not seen when looking at patients who had lower IgE levels on top (IgA^lower^IgE^lower^, md=40 years). This may be attributed to IgE levels, that often drop with higher age, possibly due to the decrease of sensitization (51). Further work on the effect of age on the combined role of IgA and IgE levels is needed.

To conclude, our results show that reduced immunoglobulin levels are common in CSU and associated with autoimmunity. Our findings implicate that especially the combination of lower serum IgA and IgE levels is of relevance in the diagnostic workup of CSU patients, as it is connected to higher rates of type IIb autoimmunity, according to BAT results, as well as more angioedema and lower basophil counts. Lower IgE, independent of IgA values, is connected to lower eosinophil counts, higher levels of IgE-anti-TPO and autoimmune phenomena, whereas lower IgA is connected to lower IgG levels and decreased quality of life. The pathomechanisms underlying the decrease in immunoglobulin levels and their interaction in CSU (e.g. impaired class switch) are still not well understood and further work on the role of both immunoglobulins as diagnostic biomarkers is required. The assessment of serum IgA and IgE levels is cost-effective and easy-to-perform. It may be useful in diagnosing subgroups of CSU (e.g. type IIb autoimmunity). Also, it may help to identify appropriate and optimal therapies for each patient based on a precision medicine approach, such as anti-IgE-, anti-interleukin-5-targeted therapies or immunosuppressives, if CSU first-line-treatment fails.

## Data Availability Statement

The raw data supporting the conclusions of this article will be made available by the authors, without undue reservation.

## Ethics Statement

The studies involving human participants were reviewed and approved by Ethikkomission, Ethikausschuss am Campus Charité - Mitte, Charité - Universitätsmedizin Berlin. Written informed consent to participate in this study was provided by the participants’ legal guardian/next of kin.

## Author Contributions

MS and KK substantially contributed to the conception and design of the study, performed analyses and interpretation of the data and drafted the manuscript. PK contributed to the statistical analyses and interpretation of the data. MS and Y-KX performed the cellular assays. JS and SF substantially contributed to the development and interpretation of laboratory tests. MMa and MMe contributed to interpretation of the data. JS, SF, PK, Y-KX, FS, SA, MMa, and MMe provided critical input to the manuscript. All authors contributed to the article and approved the submitted version.

## Funding

This study was funded by intramural grants only. MS was supported by a medical doctoral research stipend funded by Charité and Berlin Institute of Health. PK was supported by the ‘‘Russian Academic Excellence Project 5-100” and a GA²LEN stipend.

## Conflict of Interest

PK received personal fees from Novartis and Roche, outside the submitted work. FS received grants and/or personal fees from Allakos, Blueprint, Hyphens, Genentech, Novartis, Pediapharm, and Uriach, outside the submitted work. SA received grants and/or personal fees from Allakos, AstraZeneca, CSL Behring, Moxie and Sanofi, outside the submitted work. MMa received grants and/or personal fees from Allakos, Amgen, Aralez, Argenx, AstraZeneca, Celldex, Centogene, CSL Behring, FAES, Genentech, GIInnovation, Innate Pharma, Kyowa Kirin, Leo Pharma, Lilly, Menarini, Moxie, Novartis, Roche, Sanofi/Regeneron, Third HarmonicBio, UCB, and Uriach, outside the submitted work. MMe received personal fees from Amgen, Aralez, Argenx, Bayer, Moxie, Novartis, Roche, Sanofi and Uriach, outside the submitted work. KK received grants and/or personal fees from Bayer, Berlin Chemie, CSL Behring, Moxie, Novartis, Roche and Shire/Takeda, outside the submitted work.

The remaining authors declare that the research was conducted in the absence of any commercial or financial relationships that could be construed as a potential conflict of interest.

## Supplementary Material

Supplementary Figure 1 Gating strategy of the basophil activation test by flow cytometry. Relationships between the plots are indicated by arrows. Representative plots of basophils from a positive and negative control as well as after treatment with an exemplary CSU serum are shown.

## Abbreviations

ANA, Anti-nuclear antibodies; ANCA, Anti-neutrophil cytoplasmic antibodies; ASST, Autologous serum skin test; BAT, Basophil activation test; BSA, Bovine serum albumin; CSU, Chronic spontaneous urticaria; DLQI, Dermatology life quality index; EDTA, Ethylenediaminetetraacetic acid; FBS, Fetal bovine serum; IgA, Immunoglobulin A; IgE, Immunoglobulin E; IgG, Immunoglobulin G; IgM, Immunoglobulin M; IQR, Interquartile range; Md, Median; PBS, phosphate-buffered saline; SIgAD, Selective immunoglobulin A-deficiency; TBS, Tris-buffered saline; TPO, Thyroid peroxidase; UAS, Urticaria activity score.
