# Supplementary figures and images for: Lower IgA Levels in Chronic Spontaneous Urticaria Are Associated With Lower IgE Levels and Autoimmunity
Source: Front Immunol. 2021 May 3;12:657211. doi: 10.3389/fimmu.2021.657211 (PMC8128143; doi:10.3389/fimmu.2021.657211)

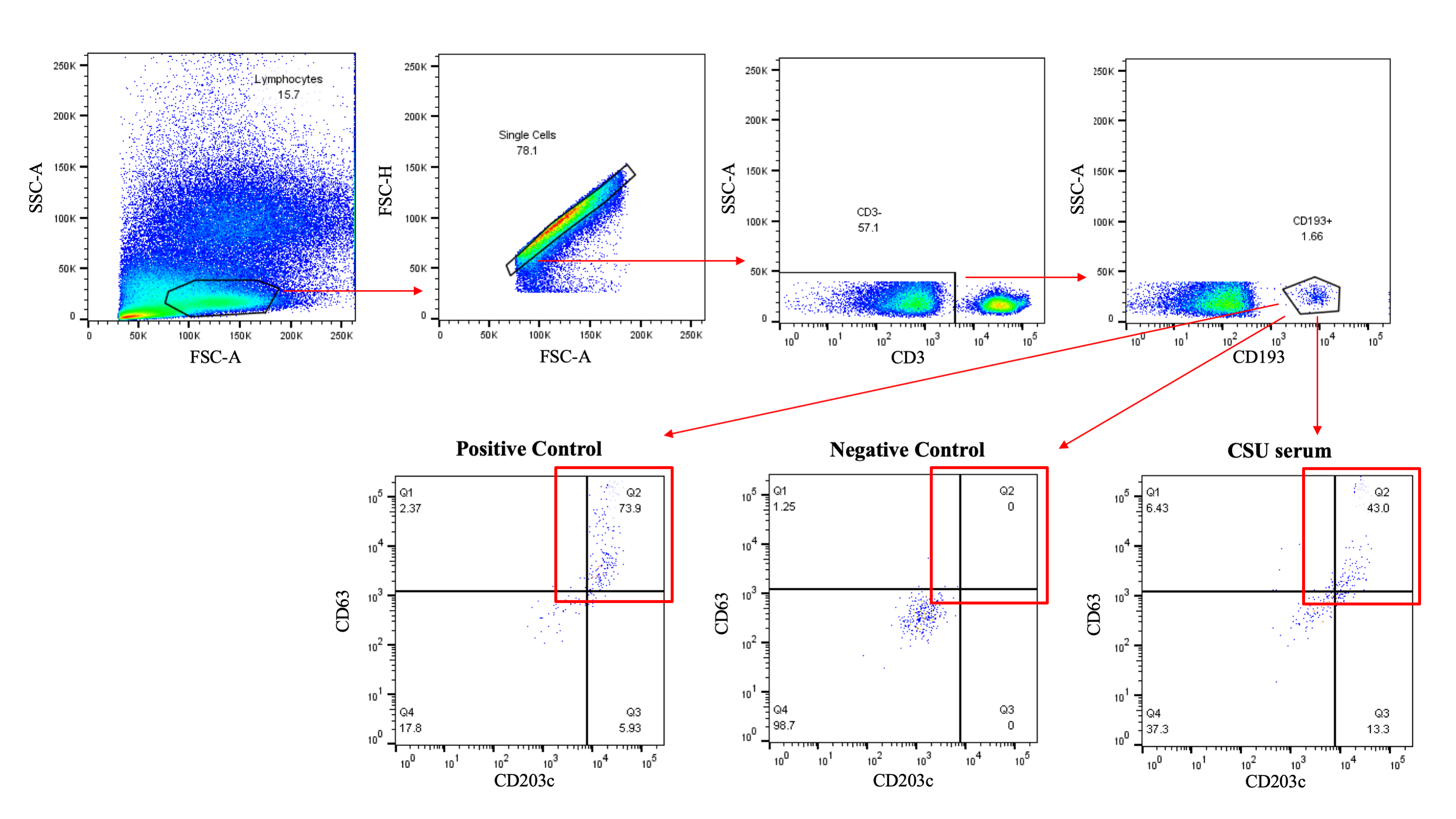

Supplement: Supplementary Figure 1 — Gating strategy of the basophil activation test by flow cytometry. Relationships between the plots are indicated by arrows. Representative plots of basophils from a positive and negative control as well as after treatment with an exemplary CSU serum are shown. [file Image_1.tiff]
